# A Giant-Cell Lesion with Cellular Cannibalism in the Mandible: Case Report and Review of Brown Tumors in Hyperparathyroidism

**DOI:** 10.1155/2017/9604570

**Published:** 2017-02-09

**Authors:** Lorenzo Azzi, Laura Cimetti, Matteo Annoni, Diego Anselmi, Lucia Tettamanti, Angelo Tagliabue

**Affiliations:** ^1^Department of Surgical and Morphological Sciences, University of Insubria, ASST dei Sette Laghi, Unit of Oral Pathology, Dental Clinic, Varese, Italy; ^2^Department of Surgical and Morphological Sciences, University of Insubria, ASST dei Sette Laghi, Unit of Pathologic Anatomy, Varese, Italy; ^3^Department of Surgical and Morphological Sciences, University of Insubria, ASST dei Sette Laghi, Unit of General Surgery 1, Varese, Italy; ^4^Meditel, Medical Centre, Unit of Radiology, Saronno, Italy

## Abstract

A small radiolucent area in the mandible was discovered in a 58-year-old woman with no oral complaints. The patient's history included only hypertension. The lesion was considered as an inflammatory cyst and was enucleated. Three months later, a CT revealed the presence of a cyst-like lesion in the mandible with thin expanded buccal cortical plate, localized erosion, and a polylobate appearance on the lingual aspect of the cortical plate. The histological diagnosis of the lesion was central giant-cell granuloma (CGCG). The lesion was thoroughly enucleated. Nevertheless, another X-ray carried out six months later revealed multiple bilateral osteolytic areas throughout the jaw. In addition, widespread cortical plate erosion was observed, as well as signs of root resorption and periodontal enlargement. There was no sign of neurological involvement, although the nerves appeared to be dislocated. After full blood chemistry analysis and detailed collection of radiographs, the final diagnosis was brown tumors in primary hyperparathyroidism. This case report demonstrates how dental clinicians may be the first-line specialists who identify a complex systemic disease before other clinicians. Finally, it highlights the role of cellular cannibalism in predicting the clinical aggressiveness of brown tumors as well as in other giant-cell lesions.

## 1. Introduction

General dentists commonly believe that small, homogeneous, radiolucent areas, which are sometimes observed during X-ray examinations, are almost certainly odontogenic cysts.

Indeed, cleavage and enucleation are only occasionally followed up with histopathological analysis. Although odontogenic cysts are very common lesions in routine practice, with radicular cysts accounting for more than 50% of cases, it should be remembered that many other diseases can affect the jaws, some more frequently than others [1].

Some of these lesions might be more aggressive, with a multilocular shape, root resorption, cortical expansion, and thinning, as well as multiple radiolucencies at times.

By way of an example, keratocystic odontogenic tumor (KCOT) [2, 3], aneurysmal bone cysts [4], intrabony haemangiomas [5], florid cemento-osseous dysplasia [6], Paget's disease [7], Langerhans cells histiocytosis [8, 9], and multiple myeloma may be detected in the jaws.

In addition, local metastasis from a primary carcinoma arising in a cyst, a primary carcinoma of the bone [10], or distant metastasis from unknown primary sites [11] can also occur within the jaws.

Even though most of the pathological entities mentioned above are very rare individually, it should be remembered that it is not uncommon to detect one of them in clinical practice.

Besides, detailed clinical and histopathological evaluation is imperative and must be performed when a radiolucency is detected by chance during an X-ray examination, even if it strongly resembles an odontogenic cyst.

Moreover, multidisciplinary diagnostic work may be required to further investigate whether there are any systemic implications or misdiagnosed malignancies underlying an osteolytic lesion of dubious origin.

This paper aims at reporting a case of an apparently innocuous cyst-like lesion of the mandible in a middle-aged woman and the subsequent diagnostic and therapeutic management which required the involvement of a multidisciplinary team.

## 2. Case Presentation

A 58-year-old woman with no oral complaints visited our dental surgery for a routine check-up.

**Figure 1:**
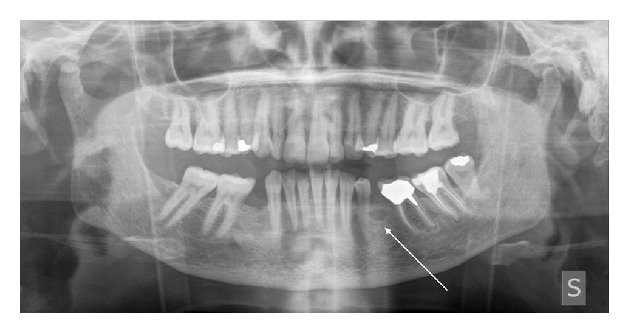
Initial clinical manifestation. A small radiolucent area (arrow) is revealed near mobile dental element 34. Chronic periodontitis is present.

**Figure 2:**
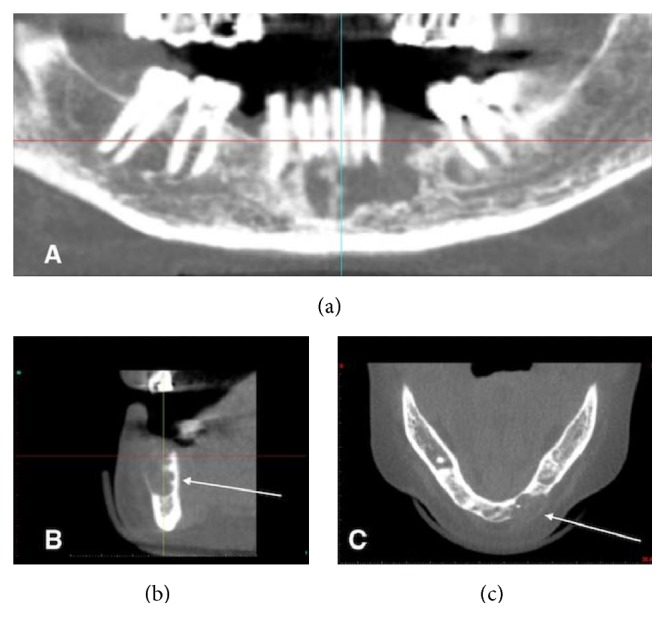
Relapse of the lesion. (a) The next examination, three months later, revealed the presence of a radiolucent cyst-like lesion in the mandible with mildly defined and irregular borders. (b) The CT radial view showed lingual cortical plate with a polylobate appearance and erosion of the buccal cortical plate (arrow). (c) The CT axial view highlighted the buccal erosion of the cortical plate (arrow).

Chronic periodontitis was detected, associated with mobility of the left permanent mandibular first premolar. The orthopantomogram only showed a small radiolucent area adjacent to the dental element (Figure 1). The patient's history only included hypertension with the use of an ACE inhibitor and a diuretic drug. The tooth was extracted and the patient was seen again three months later to evaluate the bone healing process, with a view to fitting a dental implant after a full dental-hygiene program. The CT scan revealed the presence of a radiolucent cyst-like lesion in the mandible with thin expanded buccal cortical plate and localized erosion and a polylobate appearance on the lingual aspect of the cortical plate (Figures 2(a)–2(c)). The left permanent mandibular second incisor and canine gave a negative result during pulp testing. The teeth were extracted and the bony lesion underwent thorough curettage.

**Figure 3:**
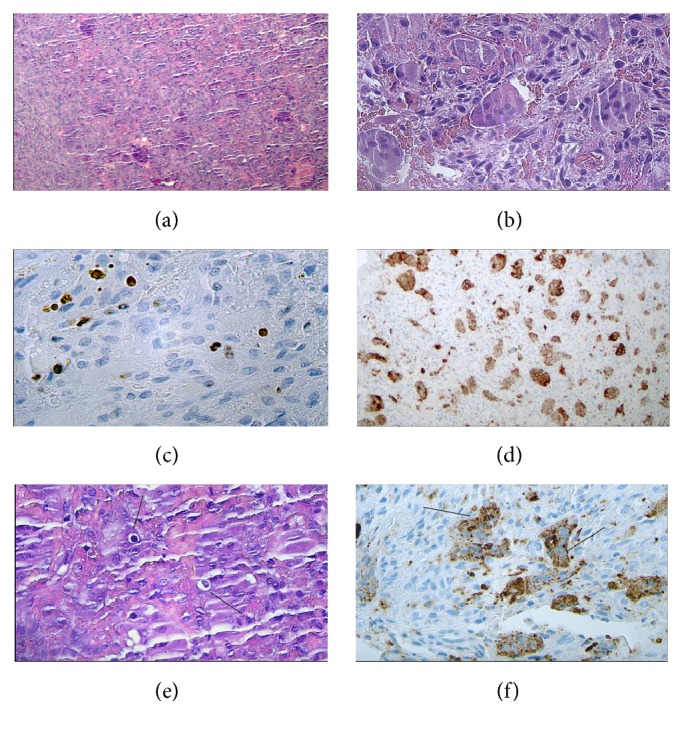
Central giant-cell granuloma (CGCG). (a) A lobulated mass of proliferating connective tissue containing many giant cells. (b) A larger view of the giant cell containing more than ten nuclei each. (c) Ki-67 indicates a low mitotic index. The lesion is defined as locally aggressive but cannot be considered as a true neoplasm. (d) CD68 antibody indicates the giant cells deriving from the monocytic-macrophagic line. (e) The so-called “cannibal cells” (arrows) indicate an aggressive CGCG and could suggest likely recurrence. (f) A larger view of the monocytic-derived cannibal giant cells highlighted with CD68.

The histological description of the sample reported the presence of an aggressive lesion with widely distributed giant cells (Figures 3(a)–3(d)). Moreover, several giant cells displayed scattered cannibalistic processes, in the absence of bizarre figures like those reported in complex cannibalism of OSCC (Figures 3(e) and 3(f)).

The pathologist provided the diagnosis of central giant-cell granuloma (CGCG).

**Figure 4:**
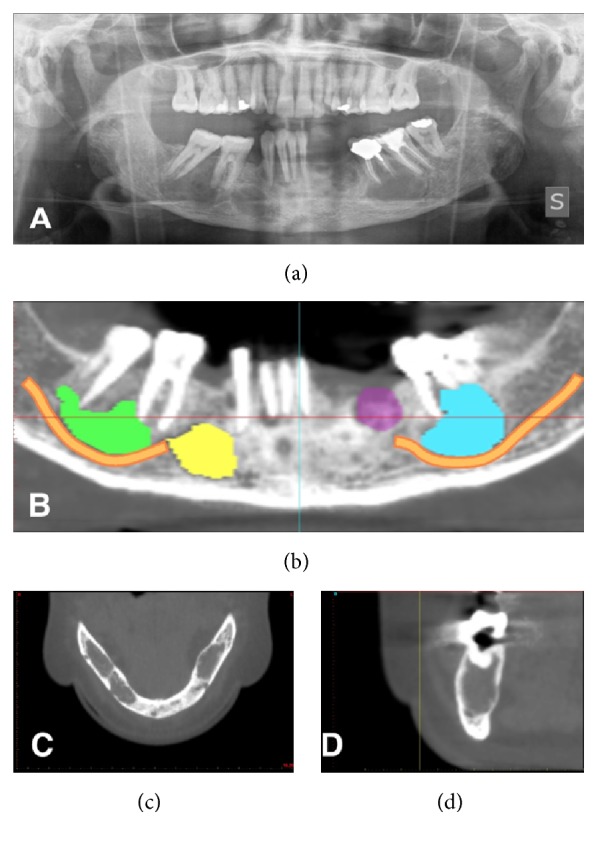
Radiographic appearance after six months. (a) Multiple osteolytic areas involving the mandible bilaterally revealed by another orthopantomogram (b–d) and CT scan six months after.

Nevertheless, another X-ray carried out six months later revealed multiple bilateral radiolucencies throughout the jaw, in both the molar and the premolar regions. In addition, widespread cortical plate erosion was observed and the left permanent mandibular first molar showed signs of root resorption, while the right permanent mandibular second molar showed periodontal ligament enlargement. There was no sign of neurological involvement, although the nerves appeared to be dislocated (Figures 4(a)–4(d)).

### 2.1. Differential Diagnosis

Given the latest clinical and radiographic presentation of the lesion, several pathological entities were considered for differential diagnosis.

#### 2.1.1. Keratocystic Odontogenic Tumor (KCOT)

KCOT is associated with frequent multiple relapses of the lesion after unsuccessful complete curettage, but the histological predominance of giant cells within the pathologic tissue excluded this hypothesis [2].

#### 2.1.2. Aggressive or Malignant Ameloblastoma

The same consideration was made as regards an aggressive variant of ameloblastoma, since its histological appearance is completely different, even if its radiographic behavior can be similar [12].

#### 2.1.3. Aneurysmal Bone Cyst

Aneurysmal bone cysts may contain many giant cells but show far more cellular pleomorphism, hyperchromatism, and a variety of other cells often of indeterminate appearance [4].

#### 2.1.4. Fibroosseous Lesions

Fibrous dysplasia may present some giant-cell foci, but its radiographic appearance, histological features, and behavior are distinctive [13].

Giant-cell lesions can be present in Paget's disease, but in this case there was no sign of pagetoid bone [7].

#### 2.1.5. Osteosarcoma

Osteosarcoma contains numerous giant cells but displays far more cellular pleomorphism, hyperchromatism, and a variety of other cells often of indeterminate appearance [14].

#### 2.1.6. Metastasis from a Distant Malignancy

Metastasis from a misdiagnosed primary malignancy could arise in the jaw, but this is rare and should produce neurological complications in the early stages, with more aggressive destructive behavior in the jaws and alveolar ridges. Histological analysis could be used to determine the primary tissue from which the malignancy originated [11].

### 2.2. Clinical Management

The multiple lesions described in Figures 4(a)–4(d) required maxillofacial evaluation for surgery, but a decision was made to verify whether the pathology was limited to the mandible or whether other body areas were involved. As the patient had had X-rays of other areas of the body during the same period, they were collected together and compared.

**Figure 5:**
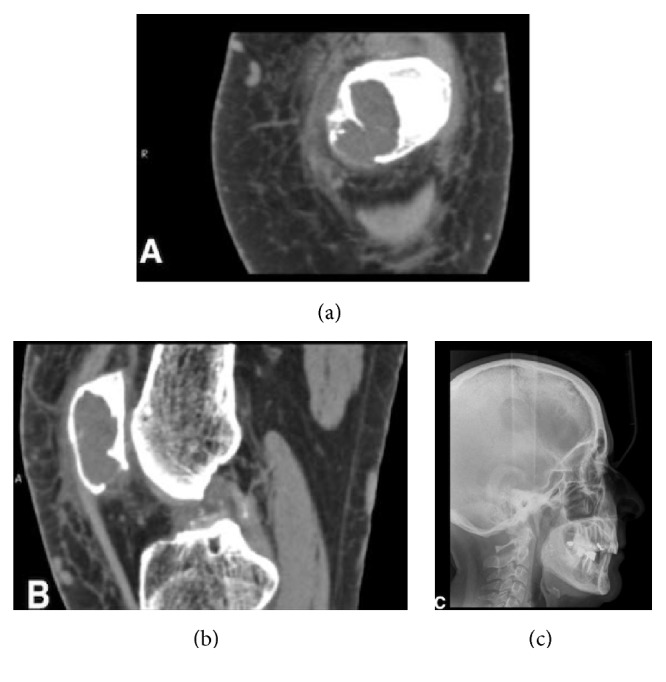
Systemic correlation. (a and b) A CT scan of the left knee highlighted the presence of an osteolytic, cyst-like, polylobate lesion which resembles those encountered in the mandible. (c) “Salt-and-pepper” appearance of the cranial vault.

MRI and CT scans of the patient's left knee revealed the presence of a cyst-like, polylobate lesion in the kneecap, with regular borders, cortical thinning, and localized erosion (Figures 5(a) and 5(b)). The radiologist affirmed that the lesion was very similar to those discovered in the mandible. Furthermore, chondrocalcinosis was noted in the right shoulder. The patient reported asthaenia, muscular tension, and mild depression. It was advisable to complete the head examination by prescribing an X-ray of the cranial vault, which showed multiple osteolytic areas with a salt-and-pepper appearance (Figure 5(c)).

A full blood chemistry analysis, including a full range of bone metabolism parameters, was prescribed. The alkaline phosphatase levels were found to be raised (374 U/L) and mild anaemia was present (RBC 3.51 10^6^/*μ*L; Hb 10.1 g/dL; HCT 31.6%). However, the most important alterations involved the parathormone (PTH 1014 pg/mL) and calcium (13.1 mg/dL) levels (Table 1).

With this data, the final diagnosis was brown tumors, with suspected primary hyperparathyroidism. Due to hypercalcemia, the patient was admitted immediately and underwent perfusion with saline water to prevent the risk of complications to the neurological and cardiovascular districts. Once hypercalcemia was treated, a neck ultrasound revealed the presence of a nonhomogeneous tumefaction involving the left inferior parathyroid gland, while the other glands were normal in size and trophism. This finding ruled out the presence of primary parathyroid hyperplasia. Primary hyperparathyroidism is often associated with adenoma, but also a functional carcinoma could not be ruled out until histopathological evaluation.

**Figure 6:**
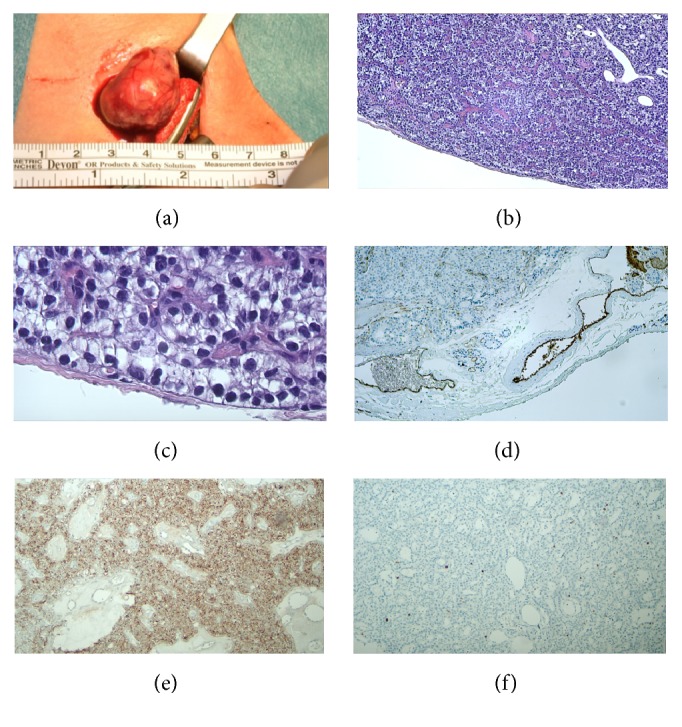
Parathyroid adenoma. (a) The patient underwent minimally invasive video-assisted parathyroidectomy (MIVAP) with intraoperative PTH determinations (baseline and 20 minutes after tumor removal) and continuous intraoperative nerve monitoring. A left inferior parathyroid adenoma was successfully removed. The baseline PTH measurement was 678 pg, and 20 minutes after excision the PTH serum level was 47 pg. Postoperative progress was uneventful. The patient was discharged the day after the operation with the appropriate prescriptions. (b and c) Parathyroid adenoma with clear cells at a low and high grade of amplification; a thin fibrous capsule is present. (d) CD34 endothelial staining showed no evidence of angioinvasion. (e) PTH staining confirmed the functional activity of the tumor. (f) Ki-67 expression was low, confirming the benign nature of the tumor.

General surgeons proceeded to remove the parathyroid lesion, which received a histopathological diagnosis of atypical parathyroid adenoma (Figures 6(a)–6(f)).

**Figure 7:**
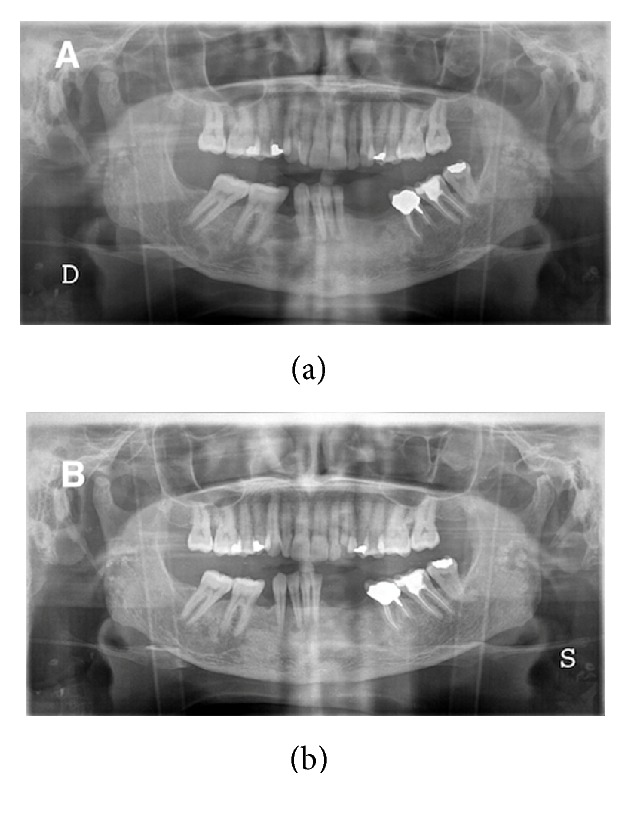
Radiographic appearance after four months and two years. (a) Signs of bone healing after four months after surgical excision of the parathyroid adenoma. (b) Complete recovery of the bony tissue two years after the operation.

Another two orthopantomograms carried out 4 months and 2 years later, respectively, showed advanced and complete healing of the bony lesions in the mandible (Figure 7). The patient was able to avoid maxillofacial surgery with resection, which would not have resolved the problem, since the origin of the condition was primary hyperparathyroidism.

## 3. Discussion

Hyperparathyroidism is defined as excessive parathyroid hormone (PTH) production. PTH is normally produced by the parathyroid glands in response to a decrease in serum calcium levels. Hyperparathyroidism can be classified as primary, secondary, and, according to several authors, tertiary.

Primary hyperparathyroidism is uncontrolled PTH production not associated with feedback regulation of the serum ionized calcium level. Most patients with primary hyperparathyroidism are over 60 years old [15]. Women are two to four times more likely to have this condition than men. The condition is typically identified during routine serological testing, and the majority of patients are relatively asymptomatic.

### 3.1. Aetiology

Primary hyperparathyroidism is usually the result of parathyroid adenoma (80–90% of cases), parathyroid hyperplasia (10–15% of cases), or, very rarely, functional parathyroid carcinoma (1% of cases).

Parathyroid adenoma is usually limited to a single gland, with a fibrous capsule generally separating it from the surrounding normal gland tissue, which could appear atrophic in some instances.

Microscopically, the adenoma can be composed of chief cells, oxyphilic cells, or clear cells.

Parathyroid hyperplasia can be divided into chief-cell hyperplasia and clear-cell hyperplasia.

Chief-cell hyperplasia is usually linked to type 1 or 2a multiple endocrine neoplasia (MEN) and is characterized by an increase in volume of all four parathyroid glands. On the contrary, the less frequent clear-cell hyperplasia is not hereditary and it is not linked to MEN. It is characterized by an enormous increase in volume of the glands, due to the combination of hyperplasia and hypertrophy involving the clear cells.

Parathyroid carcinoma is rare and can be differentiated from an adenoma since it shows a trabecular structure and spindle-shaped neoplastic cells, as well as a high mitotic index. It is aggressive and leads to cervical lymph node enlargement and vocal cord paralysis. It can sometimes be a functional carcinoma, producing PTH [16].

Independently of the three abovementioned conditions, the result is an uncontrolled increase in PTH serum levels, which is defined as primary hyperparathyroidism.

### 3.2. Clinical Features

Primary hyperparathyroidism is a complex condition which involves several body districts and requires a multidisciplinary approach both for diagnosis and for treatment.

The increased serum PTH level leads to excessive osteoclastic activity, with the release of calcium, phosphorus, and hydroxyproline due to the generalized demineralization of the bone district.

The clinical signs and symptoms of primary hyperparathyroidism are the expression of the main feature of the pathology, hypercalcemia. The increased calcium level may cause salt to be deposited in the soft tissues, with the consequent involvement of sclera (band keratitis), tendons (calcific tendonitis), joint cartilage (chondrocalcinosis and arthralgia), kidney parenchyma (nephrocalcinosis), and the pancreas.

PTH stimulates calcium tubular resorption, but serum levels exceeding 12 mg/dL lead to the onset of hypercalciuria. On the other hand, PTH promotes phosphorus renal elimination, and so hypophosphaturia and hypophosphataemia are observed. Kidney stones are the most common sign of kidney involvement in primary hyperparathyroidism. Furthermore, PTH increases calcium absorption in the bowel due to the augmented conversion of 25(OH) D into 1,25(OH)2 D.

As a result, hypercalcemia is maintained, as mentioned above, by increased bone resorption, increased calcium absorption in the bowel, and inadequate kidney elimination of calcium.

Hypercalcemia leads to functional alterations in the central nervous system, with ideation disorders, recent memory loss, emotional lability, and sleepiness.

In severe cases, a state of confusion, drowsiness, and coma may occur.

Other clinical signs and symptoms of hypercalcemia are intense asthaenia and fatigue, especially along the proximal musculature of the limbs, vomiting, epigastric pain, duodenal ulcer, hypertension, and, in the case of hypercalcemic crisis, bradycardia and heart failure.

A variety of osseous changes may occur in conjunction with hyperparathyroidism. One of the first clinical signs of the disease can be seen during radiographic examination, in the subperiosteal resorption of the phalanges of the index and middle fingers. Generalized loss of the lamina dura surrounding the roots of teeth is also seen as an early manifestation of the condition. Alterations in trabecular pattern characteristically develop next. A decrease in trabecular density and blurring of the normal trabecular density occur, often resulting in a “ground glass” appearance.

### 3.3. Brown Tumors, Giant-Cell Lesions, and Cellular Cannibalism

With persistent disease, other osseous lesions develop, such as the so-called brown tumors of hyperparathyroidism [17]. These lesions derive their name from the colour of the tissue specimen, which is usually dark red-brown because of the abundant haemorrhaging and haemosiderin deposits within the tumor. These lesions appear radiographically as well-demarcated unilocular or multilocular radiolucent patches. They commonly affect the mandible, clavicle, ribs, and pelvis. They may be solitary but are often multiple, and long-standing lesions may produce significant cortical expansion. The most severe skeletal manifestation of chronic hyperparathyroidism is known as osteitis fibrosa cystica (Von Recklinghausen's disease of bone [18]), a condition that develops from the central degeneration and fibrosis of long-standing brown tumors. Brown tumors cannot be reliably distinguished from nonendocrine central giant-cell granulomas (CGCGs).

CGCGs of the jaw are uncommon. They account for 0.17% of oral biopsies reported by Waldron and Shafer [19]. They are more common in women, with a 3 : 1 F : M ratio. It should be considered that they are most frequent in patients under 30s, and this feature is useful to distinguish them from brown tumors. The jaw is the most common site. They were first described by Jaffe as giant-cell reparative granulomas [20], as they were thought to represent a reparative process.

However, it has now been ascertained that there is no granuloma formation in the histological sense, and they are not linked to a reparative process after trauma or cyst removal. On the contrary, they may grow rapidly and be mildly destructive instead of reparative. In 40% of cases, CGCGs show an aggressive behavior such as perforation of the cortical bone and root resorption. One distinctive histological feature is the cannibalistic component among the giant cells in the lesion [21]. This variant is known as aggressive CGCG and may have been a suitable diagnosis in this case.

Cellular cannibalism is defined as a large cell enclosing a slightly smaller one within its cytoplasm and is a characteristic morphological feature exclusively seen in aggressive malignancies, such as breast carcinoma, giant-cell carcinoma of the lung, gall bladder carcinoma, endometrial stromal carcinoma, malignant thymoma, and malignant melanoma [22]. It is well correlated with the aggressiveness, degree of anaplasia, invasiveness, and metastatic potential of the malignancy.

Sarode et al. were the first to describe cellular cannibalism in oral squamous cell carcinoma. Besides, they reported bizarre morphological appearances of cannibalism, wherein one malignant cell was engulfing the other and this complex was further engulfed by another cell or one cell was engulfing two cells at a time. This kind of feature was named complex cannibalism [23].

Cannibalism in malignant tumor is caused due to a shift in the metabolic pathway that encourages selection of certain cell phenotypes that are able to survive in the caustic environment. These selected malignant cells are highly virulent and cannibalize other malignant cells to survive and progress in adverse conditions within the microenvironment such as hypoxia, low nutrient supply, and acidity.

However, cellular cannibalism has been reported in a benign tumor called the giant-cell tumor of the tendon sheath [24].

A recent study by S. C. Sarode and G. S. Sarode demonstrated that mean cannibalistic giant-cell frequency was greater in aggressive CGCG compared to nonaggressive CGCG. Thus, the presence of cannibalistic giant cells can be used to predict the biological behavior of giant-cell lesions [21].

The biological behavior of CGCG of the jaw ranges from a quiescent lesion with the absence of symptoms, no root resorption or cortical perforation, slow growth, and low recurrence rate (nonaggressive CGCG) to an aggressive pathological process, characterized by pain, rapid growth, root resorption, cortical perforation, and high tendency to recur (aggressive CGCG).

The mean cannibalistic giant-cells frequency was greater in aggressive CGCG than in nonaggressive CGCG. Thus, it can be used for predicting the biological behavior of CGCG.

In giant-cell lesions, the cannibalistic cells are derived from a monocyte-macrophage lineage and resemble osteoclasts (CD68+). Thus, these cells possess the inherent property of engulfment, which is responsible for the cannibalism of stromal tumor cells and represents high metabolic activity in giant-cell lesions [22].

In this case report, we presented a case of cellular cannibalism within brown tumors of the mandible.

The cannibalistic activity looked classic and the lesions showed the same features of an aggressive CGCG: root resorption, cortical expansion, periodontal enlargement, and multiple relapses. The presence of a cannibalistic activity within the histologic sample confirmed the presence of clinical aggressiveness of the lesion and the relapse which followed surgical excision.

### 3.4. Diagnosis

Primary hyperparathyroidism is diagnosed by means of blood chemistry testing: raised PTH, hypercalcemia, hypophosphataemia, hyperuricaemia, anaemia, raised ESR, hypomagnesaemia, hypokalaemia, and hyperchloremic acidosis.

An X-ray of the urinary tract can reveal the presence of kidney stones or minor calcifications involving the renal parenchyma.

During the later stages, cranial X-rays show alterations within the bones of the cranial vault, which reveal focal areas of demineralization as opposed to sclerotic sites. This is the so-called salt-and-pepper skull.

Ultimately, it is necessary to identify the primary parathyroid disorder: ultrasound, CT, and ^201^Tl and ^99^Tc double-track scintigraphy are used for this purpose [25].

If a parathyroid adenoma is not found, suspected PTH-like peptide (PTHrP) production in a paraneoplastic syndrome must be taken into consideration [26].

### 3.5. Management and Treatment

Treatment involves surgical excision of the affected parathyroid.

However, hypercalcemia must be treated with saline solution infusion, diuretics, and other symptomatic treatments to restore normal electrolyte levels.

Differential diagnoses of hypercalcemia also include vitamin D intoxication, multiple myeloma, sarcoidosis, vitamin A intoxication, Burnett syndrome (milk-alkali syndrome), thyrotoxicosis, prolonged immobilization, adrenocortical insufficiency, and Paget's disease. In all these cases, PTH levels are below normal, while hypercalcemia may be absent in several cases [27].

When an osteolytic lesion is diagnosed as a CGCG, the dental clinician should consider the differential diagnosis of brown tumor in hyperparathyroidism. Differential diagnosis is only possible by means of blood chemistry analysis, as mentioned above.

The decision to surgically excise brown tumors once hyperparathyroidism has been diagnosed should be made after enucleation of the parathyroid adenoma to prevent unsuccessful surgical procedures, which could lead to deterioration in the condition with more aggressive relapses.

However, dental clinicians are commonly thought to proceed with surgical excision following the initial histological diagnosis of CGCG.

## 4. Conclusion

This case report and review shows, on the one hand, the importance of multidisciplinary management of giant-cell lesions affecting the jaws, while, on the other hand, it demonstrates how dental clinicians may be the first-line specialists who identify a complex systemic disease before other clinicians. It is therefore essential for dental clinicians to communicate properly and promptly with other specialists in the event of a suspected systemic disease primarily expressed in the oral cavity.

Finally, the presence of cannibalistic activity in the monocyte-macrophage derived giant cells in brown tumors can be considered as a prognostic feature to predict the clinical aggressiveness of the lesion and the recurrence rate.

## Figures and Tables

**Table 1:** Blood chemistry analysis. Bone metabolism was altered, parathormone was critically raised, and the calcium levels posed a high risk of heart failure.

Investigation	Results	Normal values
ESR	26 mm	<15
Alkaline phosphatase	374 U/L	40–150
*Parathormone*	*1014* pg/mL	15–68
Calcium	13,1 mg/dL	8,4–10,2
